# Differentiation of *Trichinella* species (*Trichinella spiralis*/*Trichinella britovi versus Trichinella pseudospiralis*) using western blot

**DOI:** 10.1186/s13071-018-3244-3

**Published:** 2018-12-12

**Authors:** Maria Angeles Gómez-Morales, Alessandra Ludovisi, Marco Amati, Simona Cherchi, Daniele Tonanzi, Edoardo Pozio

**Affiliations:** European Union Reference Laboratory for Parasites, Istituto Superiore di Sanità, viale Regina Elena 299, 00161 Rome, Italy

**Keywords:** *Trichinella*, Diagnosis, Serology, Western blot, Crude worm extract, Epidemiology

## Abstract

**Background:**

Trichinellosis is a meat-borne zoonotic disease caused by parasites of the genus *Trichinella*. To date, 12 taxa have been described. The identification of *Trichinella* species is crucial in order to identify the possible source of infection, the geographical origin of the parasite and to assess risk of infection for domestic pigs and humans. Specific identification of the etiological agent is not always feasible using direct methods since the source of infection can be untraceable. The aim of this study was to develop a diagnostic tool to infer the causative *Trichinella* species using western blot patterns of sera derived from infected animal and human hosts.

**Methods:**

Sera from mice experimentally infected with *Trichinella spiralis*, *Trichinella britovi*, *Trichinella pseudospiralis* and *Trichinella papuae* were tested by western blot using homologous and heterologous crude worm extracts (CWE) and a highly sensitive detection system based on chemiluminescence. In addition, sera from pigs experimentally infected with *T. spiralis*, *T. britovi* and *T. pseudospiralis* and from patients with confirmed *T. spiralis*, *T. britovi* and *T. pseudospiralis* infections, were also included.

**Results:**

Sera from mice infected with one *Trichinella* species reacted with CWE proteins from all four investigated species. Likewise, sera derived from pigs and humans infected with one *Trichinella* species reacted with CWE proteins from all the three investigated species. Using *T. spiralis* CWE, sera from *T. pseudospiralis-*infected hosts yielded a characteristic pattern of reactivity using Wb, which differed to that produced by *T. spiralis*/*T. britovi-* or *T. papuae*-infected host sera.

**Conclusions:**

The present study suggests that western blot using *T. spiralis* CWE may be a useful tool to distinguish *Trichinella* infections caused by *T. pseudospiralis* from those caused by *T. spiralis* or *T. britovi.* This method may support epidemiological investigations, particularly when the source of infection is not traceable.

**Electronic supplementary material:**

The online version of this article (10.1186/s13071-018-3244-3) contains supplementary material, which is available to authorized users.

## Background

Trichinellosis is a meat-borne zoonotic disease caused by parasites of the genus *Trichinella.* To date, 12 taxa are known, including the encapsulating species *Trichinella spiralis*, *Trichinella nativa*, *Trichinella britovi*, *Trichinella murrelli*, *Trichinella nelsoni*, *Trichinella patagoniensis* and genotypes *Trichinella* T6, T8 and T9 exclusive to mammals, and non-encapsulating species *Trichinella pseudospiralis*, *Trichinella papuae* and *Trichinella zimbabwensis* infecting mammals and birds or mammals and reptiles [1]. The most common source of infection for humans is raw pork but raw meat and meat-derived products from other omnivores (e.g. wild boar), carnivores (e.g. bear, cougar, fox, badger, jackal, walrus and dog) and a herbivore (i.e. horse), were also identified as important sources of infections [2].

The severity of trichinellosis in humans can range from subclinical to fatal. However, given that no pathognomonic signs or symptoms exist, infections that are suspected based on the clinical picture and laboratory findings should be serologically confirmed through the detection of anti-*Trichinella* IgG [3]. Currently, ELISA as a primary screening test and Western blot (Wb) as a confirmatory assay based on excretory/secretory antigens (ESA), are the most commonly used tools to diagnose human trichinellosis [3, 4] and to monitor *Trichinella* spp. infection in animals [5–8].

Animal hosts can harbour infective muscle larvae (ML) before antibodies can be detected, therefore serological methods should not be used for the surveillance of *Trichinella* infection in food animals. Nevertheless, ELISA can be used to monitor infection in domestic pigs and wildlife populations; however, a confirmatory test, such as Wb, should be performed to confirm ELISA positive sera [9].

ESA originating from larval secretions consist of a group of structurally related glycoproteins, which contain a predominant antigen epitope recognized in animals and humans infected with *T. spiralis*, or any of the other taxa of *Trichinella* currently known [10]. Consequently, tests utilizing ESA can detect infection caused by any of the known *Trichinella* taxa, but are unable to provide species-specific identification. The identification of the *Trichinella* taxon is crucial in order to determine the possible source of infection, the geographical origin of the parasite and to assess potential risk of infection for domestic pigs and humans. Additionally, species identification of the etiological agent is not always feasible using direct methods since the source of infection can be untraceable.

Four *Trichinella* species (*T. spiralis*, *T. nativa*, *T. britovi* and *T. pseudospiralis*) are known to occur within the European Union (EU). *Trichinella nativa* is restricted to wild carnivores of arctic and subarctic regions and few cases of human infections were documented in the EU due to imported bear meat or bear meat consumed abroad [11, 12]. In contrast, *T. spiralis*, *T. britovi* and *T. pseudospiralis* are widely distributed within the continent with varying prevalence rates depending on animal species (swine *versus* carnivores) and husbandry conditions [13, 14].

The objective of the present study was to develop a diagnostic tool to differentiate between *Trichinella* species causing infection in animals and humans, based on the protein band pattern of reactivity of the host serum sample *versus Trichinella* spp. antigens using western blot.

## Methods

### Sera

Samples were collected at 45 days post-infection (dpi) from 28 CD1 mice, which had been infected with 200 larvae/mouse of *T. spiralis* (five mice), *T. britovi* (five mice), *T. pseudospiralis* (five mice), *T. papuae* (five mice), *T. nativa* (one mouse), *T. murrelli* (one mouse), *Trichinella* T6 (one mouse), *T. nelsoni* (one mouse), *Trichinella* T8 (one mouse), *Trichinella* T9 (one mouse), *T. zimbabwensis* (one mouse) and *T. patagoniensis* (one mouse). All mice had tested positive for the presence of anti-*Trichinella* IgG by ELISA and Wb using *T. spiralis* ESA. Sera were also collected 65 dpi from 15 domestic pigs, which had been infected with 10,000 larvae of *T. spiralis* (five pigs), *T. britovi* (five pigs), and *T. pseudospiralis* (five pigs) and had tested positive for the presence of anti-*Trichinella* IgG by ELISA and Wb using *T. spiralis* ESA.

A total of 14 human sera obtained from five patients with confirmed *T. spiralis* infections acquired during a horsemeat outbreak [15], five patients with confirmed *T. britovi* infections acquired during a wild boar meat outbreak [16] and four patients with confirmed *T. pseudospiralis* infections sustained during a wild boar meat outbreak [17] were included in this study. All 14 human sera had tested positive for the presence of anti-*Trichinella* IgG by ELISA and Wb using *T. spiralis* ESA. The diagnosis of trichinellosis was based on the algorithm proposed by Dupouy-Camet & Bruschi [3]. The 20 murine, 15 porcine and 14 human sera yielded the three band patterns characteristic of *Trichinella* infection using Wb [4].

### Antigens

Muscle larvae (ML) were collected from mice, which had been infected three months earlier with 500 ML of *T. spiralis* and *T. britovi*, thus representing the group of encapsulating species, and *T. pseudospiralis* and *T. papuae* illustrative of those not able to induce capsule formation. ML were collected by HCl-pepsin digestion. Following digestion, ML were washed several times using 0.1M phosphate buffered saline pH 7.2 (PBS), and were then stored at -70 °C in the presence of protease inhibitors (Sigma-Aldrich, Saint Louis, MO, USA). After four thawing/freezing cycles, ML were crushed in a glass Potter homogenizer using a Teflon pestle, and further disintegrated by sonication. The larval suspension was maintained overnight at 4 °C with magnetic stirring and centrifuged for 1 h at 13,000× *g* at 4 °C. The protein concentration of the supernatant was determined by the Bradford method. Three different CWE batches were prepared for each of the four *Trichinella* species.

### Serological methods

#### ELISA with excretory/secretory antigens

An in-house ELISA was used in accordance with previously published protocols [18, 19]. Murine, porcine and human sera were diluted 1:100, 1:50 and 1:200, respectively. Peroxidase-labelled anti-mouse IgG or anti-swine IgG was diluted 1:30,000, whereas peroxidase labelled anti-human IgG was diluted 1:50,000 (Kierkegaard and Perry Laboratories, Gaithersburg, MD, USA). The optical density (OD) was obtained by reading the reaction at 450 nm using an ELISA plate microtiter reader (Dynex Technologies, Chantilly, VA, USA).

#### Western blot with crude worm extracts

Murine, porcine and human sera were diluted 1:100 and tested by Wb using a highly sensitive detection system based on chemiluminescence [7]. Sera with an ELISA OD value higher than 1.5 were also tested at a dilution of 1:500. Briefly, 150 μg of *T. spiralis*, *T. britovi*, *T. pseudospiralis* and *T. papuae* CWEs were diluted and loaded in 10% pre-cast NuPage Novex Bis-Tris Gels® (Life Technologies, Carlsbad, CA, USA) as reported in the instructions for electrophoresis using the XCell SureLock® Mini-Cell (Life Technologies). Proteins were electrophoretically separated under reducing conditions and transferred to nitrocellulose (Bio-Rad, Hercules, CA, USA) at RT for 1 h. The nitrocellulose filters were blocked with 5% skimmed milk in 1× Tris Borate Saline Tween (TBST, 50 mM Tris pH 8.0, 150 m NaCL, 1% Tween 20) at 4 °C overnight and washed three times with 1× TBST. Nitrocellulose filters with *T. spiralis* CWE were cut into strips, each of which was then incubated with 1:200 mouse, 1:500 porcine or 1:400 human sera with 3% w/v skimmed milk (Sigma-Aldrich) in 1× TBST at RT for 1 h. After washing three times with 1× TBST, the strips were incubated for 1 h with a 1:2500 dilution of goat anti-mouse IgG, or a 1:3000 dilution of goat anti-pig IgG, or a 1:10,000 dilution of goat anti-human IgG, conjugated with horseradish peroxidase (Bio-Rad). To reveal proteins with high efficiency, the LiteAblot® Plus chemiluminescence system (Euroclone, Pero, Milan, Italy) was added to the strips for 5 min. The proteins were then visualized on a ChemiDoc™ XRS System (Bio-Rad) and images were analysed using the Image Lab™ software version 4.0 (Bio-Rad). Each individual serum was tested three times by Wb using three different CWE batches.

## Results

### Protein profiles of *T. spiralis*, *T. britovi*, *T. pseudospiralis* and *T. papuae* CWEs

**Fig. 1:**
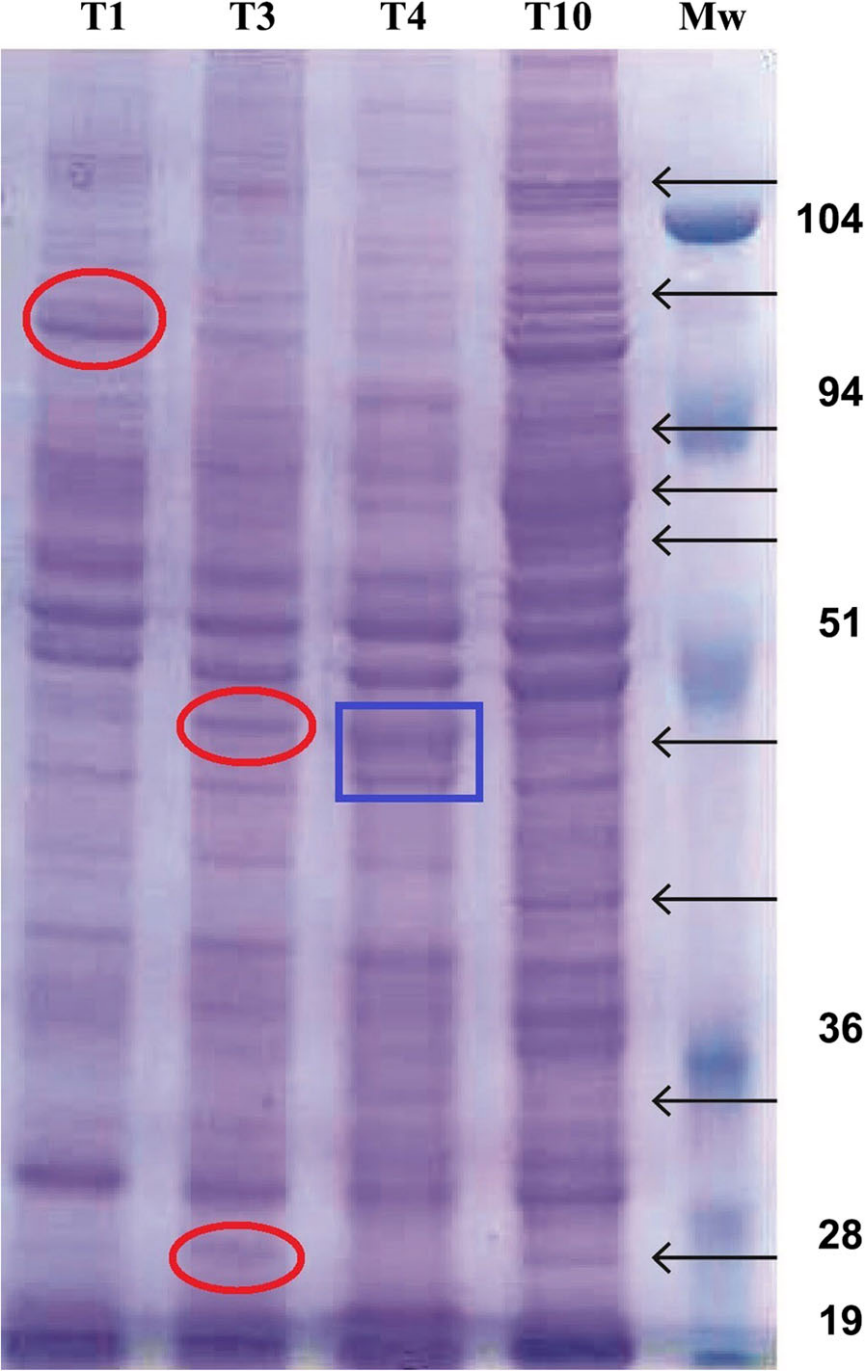
Electrophoretic protein patterns of *Trichinella spiralis*, *T. britovi*, *T. pseudospiralis* and *T. papuae* crude worm extracts by SDS-PAGE. Lanes 1–4: *T. spiralis*, *T. britovi*, *T. pseudospiralis* and *T. papuae* crude worm extracts (CWE), respectively; Lane Mw: molecular weights in kDa. Arrows show the main differences among CWE protein patterns. Red circles indicate differences in band intensity between CWE profiles of *T. spiralis* and *T. britovi*; blue box indicates differences in the protein pattern between *T. pseudospiralis* and encapsulated species (*T. spiralis* and *T. britovi*)

The protein profile patterns of the three CWE batches within the same species run by SDS-PAGE were identical (data not shown). The CWE profiles of *T. spiralis* and *T. britovi* showed some differences in band intensity: (i) a more intense 96 kDa band in *T. spiralis* CWE than in *T. britovi* CWE; and (ii) more intense 45 kDa and 23 kDa bands in *T. britovi* than in *T. spiralis* CWE (Fig. 1, red circles). Differences in the protein pattern were detected between encapsulated (*T. spiralis* and *T. britovi*) species and the non-encapsulated *T. pseudospiralis* and *T. papuae*. The protein profile of *T. papuae* appeared to be the most different from the other three CWE profiles (Fig. 1). Proteins of *T. pseudospiralis* produced a different electrophoretic pattern than that of *T. spiralis* or *T. britovi*, despite having similar molecular weights (Mws; 45 and 43 kDa; Fig. 1, blue box).

### Wb reactivity of *T. spiralis*, *T. britovi*, *T. pseudospiralis* and *T. papuae* murine sera with homologous and heterologous CWE

**Fig. 2:**
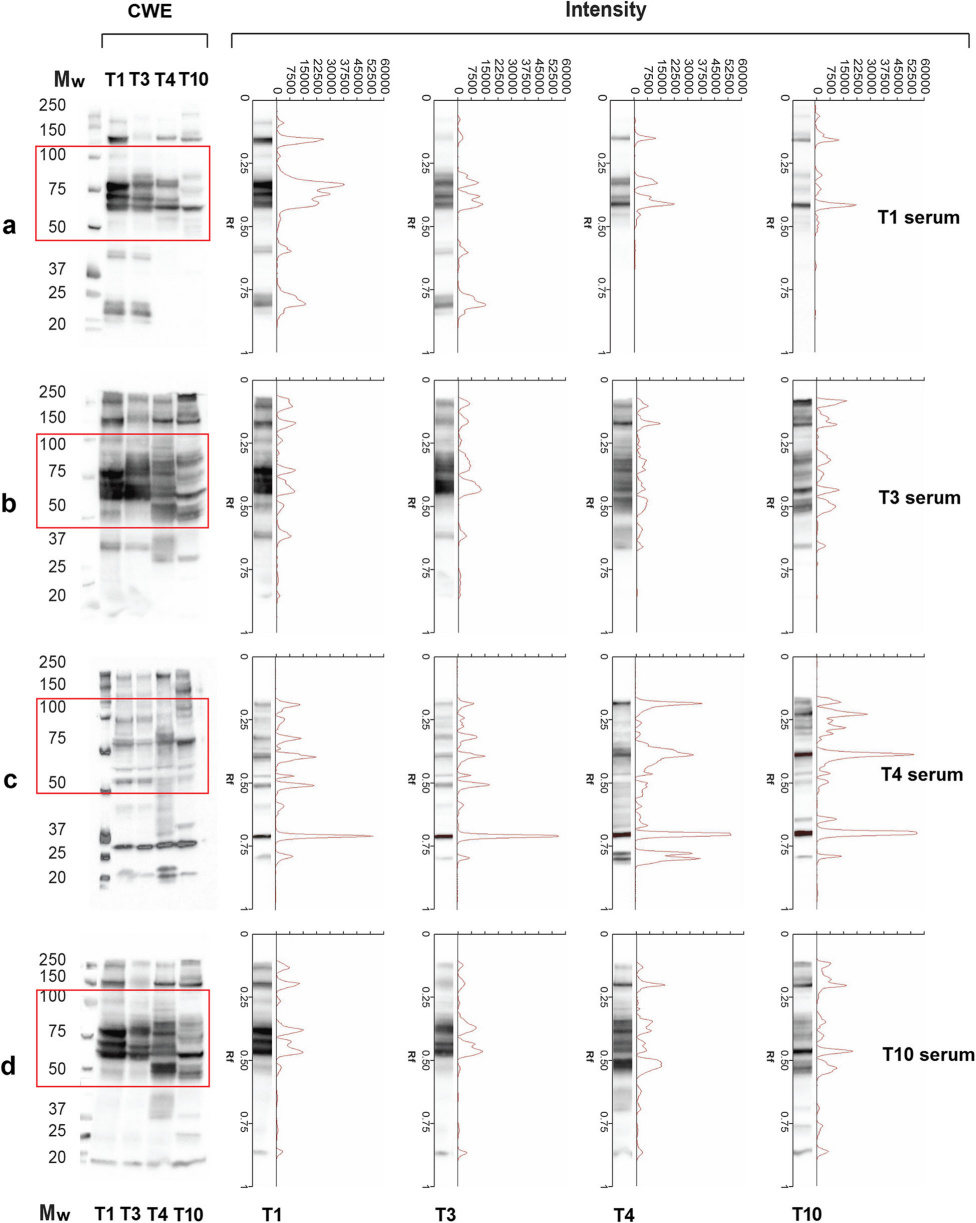
Western blot (Wb) patterns, signal intensities and relative migration (Rf) values of *Trichinella* spp. crude worm extracts (CWE) with serum samples from mice infected with *Trichinella spiralis* (Lane T1), *T. britovi* (Lane T3), *T. pseudospiralis* (Lane T4) and *T. papuae* (Lane T10)*.* **a** *T. spiralis* (T1) infected mouse serum; **b** *T. britovi* (T3) infected mouse serum; **c** *T. pseudospiralis* (T4) infected mouse serum; **d** *T. papuae* (T10) infected mouse serum. Comparison of Wb patterns and intensities are on the left and on the right, respectively. Lane Mw: molecular weights in kDa. Red boxes refer to the highest differences detected in the Wb patterns

Sera from mice infected with a given *Trichinella* species reacted with CWE from the other three species by Wb; however, the patterns of reactivity were different for both band intensity and presence/absence of bands (Fig. 2). Sera from *T. spiralis-*infected mice reacted with the four CWEs showing bands from 60 to 150 kDa. However, bands from 23 to 42 kDa were observed only when sera were blotted with *T. spiralis* and *T. britovi* CWEs (Fig. 2a, Lanes 1 and 2). The reactivity patterns of *T. spiralis* and *T. britovi* CWEs with a *T. spiralis*-infected murine serum displayed different signal intensities (Fig. 2a, Lanes 1 and 2); the pattern of reactivity of the same serum sample with *T. pseudospiralis* and *T. papuae* CWEs showed signals of different intensities for bands above 50 kDa and the absence of bands below 50 kDa (Fig. 2a, Lanes 3 and 4).

Sera from *T. britovi-* and *T. papuae-*infected mice displayed very similar reactivity patterns, when they reacted with the CWEs of encapsulated (Fig. 2b, d; Lanes 1 and 2) and non-encapsulated species (Fig. 2b, d; Lanes 3 and 4).

**Fig. 3:**
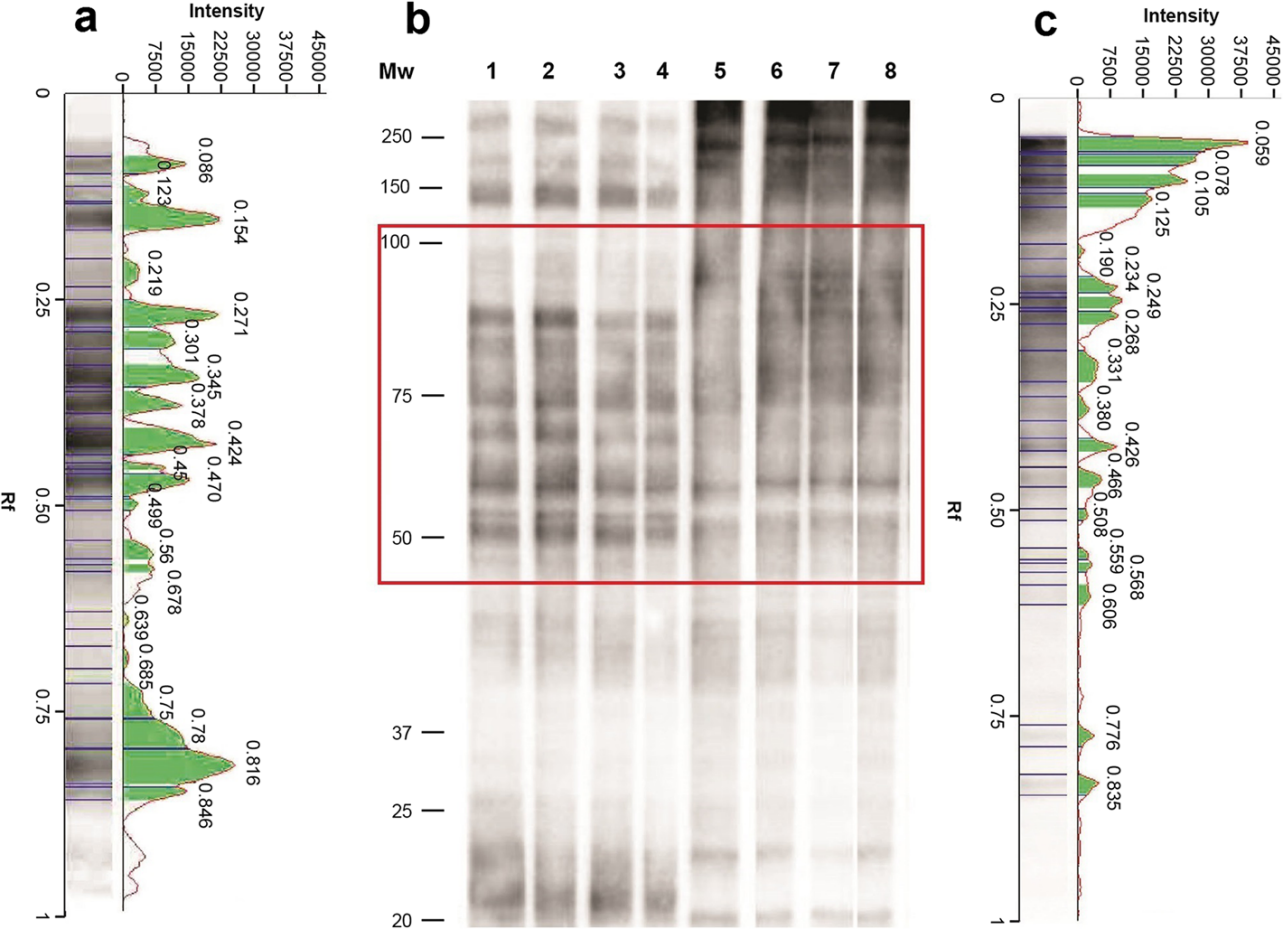
Western blot (Wb) patterns, signal intensities and relative migration values of *Trichinella spiralis* crude worm extract (CWE) with sera from infected mice. **a** Signal intensity and relative migration values of *T. spiralis* CWE with a serum sample from a *T. spiralis* infected mouse (**b**, Lane 1). **b** Lane Mw: molecular weights in kDa; Wb pattern of four sera from *T. spiralis (Lanes 1–4) or *T. pseudospiralis* (Lanes 5–8) infected mice with *T. spiralis* CWE. **c** Signal intensity of *T. spiralis* CWE with a serum sample from a *T. pseudospiralis-*infected mouse (**b**, Lane 8). The red box indicates the highest differences detected in the Wb patterns

Sera from *T. spiralis-*, *T. britovi-* or *T. papuae-*infected mice reacted with *T. spiralis* CWE showing a similar profile (Fig. 2a, b, d; Lane 1). This differed from the pattern seen for sera samples from *T. pseudospiralis-*infected mice with *T. spiralis* CWE (Fig. 2c, Lane 1). Moreover, sera from *T. pseudospiralis-*infected mice reacted with *T. spiralis*, *T. britovi* and *T. papuae* CWE proteins yielding a band pattern (Fig. 2c, Lanes 1, 2 and 4) which differed from those produced by sera of mice infected with the other species (*T. spiralis*, *T. britovi* and *T. papuae*) with the same CWE (Fig. 2a, b, d). As shown in Fig. 3, the reactivity patterns of *T. pseudospiralis* and *T. spiralis* sera with heterologous and homologous CWEs, yielded different profiles.

Looking at the relative mobility (Rf) and at the signal intensity of *T. spiralis* CWE proteins recognized by mouse sera, the highest differences were detected from 50 to 100 kDa (Fig. 3, red boxes). The Rf of *T. spiralis* CWE reacting proteins with a representative *T. spiralis* mouse serum were 0.470, 0.450, 0.424, 0.378, 0.345, 0.301, 0.271 and 0.219 mm, whereas the Rf of *T. spiralis* CWE reacting proteins with a representative *T. pseudospiralis* mouse serum were 0.508, 0.466, 0.426, 0.380, 0.331, 0.268, 0.249 and 0.234 mm. Each *T. spiralis* or *T. pseudospiralis* mouse serum sample generated a “fingerprint” of its own identity.

### Wb reactivity of sera from pigs infected with *T. spiralis*, *T. britovi* and *T. pseudospiralis* with *T. spiralis* CWE

Given that *T. spiralis* is the species generally maintained in laboratory mice and used to produce antigens for the serological detection of *Trichinella* species, we focused on the reactivity of sera from pigs infected with *T. spiralis*, *T. britovi* and *T. pseudospiralis* with *T. spiralis* CWE.

**Fig. 4:**
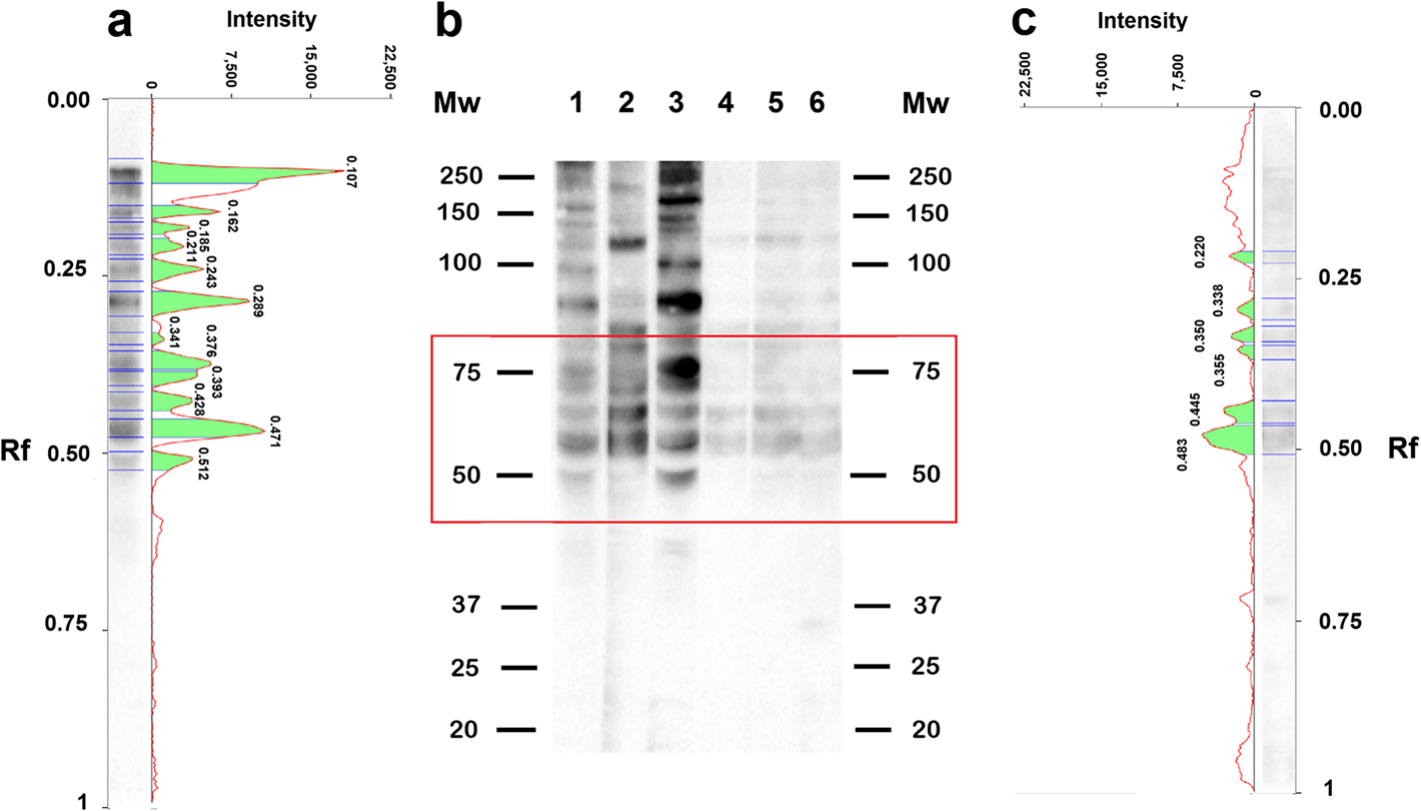
Western blot patterns, signal intensities and relative migration values of *Trichinella spiralis* crude worm extract (CWE) with sera from infected pigs. **a** Signal intensity and relative migration values of *T. spiralis* CWE with a serum sample from a *T. spiralis* infected pig (**b**, Lane 1). **b** Lane Mw, molecular weights in kDa, Wb of *T. spiralis* CWE reacting proteins with three representative serum samples from *T. spiralis* (Lanes 1–3) or *T. pseudospiralis* (Lanes 4–6) infected pigs. **c** Signal intensity and relative migration values of *T. spiralis* CWE with a serum sample from a *T.pseudospiralis* infected pig (**b** Lane 6). The red box indicates the highest differences detected in the Wb patterns

Sera from *T. spiralis-* and *T. pseudospiralis-*infected pigs reacted with *T. spiralis* CWE showing different protein profiles mainly in the range of 50 to 75 kDa. In this Mw range, the Rf of *T. spiralis* CWE reacting proteins with three representative *T. spiralis-*infected pig sera were 0.471, 0.428, 0.393, 0.376, 0.341, 0.289 and 0.243 mm, whereas the Rf of *T. spiralis* CWE reacting proteins with three representative *T. pseudospiralis-*infected pig sera were 0.483, 0.445, 0.355, 0.350, 0.338 and 0.220 mm (Fig. 4, red box). Sera from *T. britovi-* and *T. spiralis-*infected pigs showed the same reactivity pattern with *T. spiralis* CWEs (data not shown).

### Wb reactivity of sera from patients infected with *T. spiralis*, *T. britovi* and *T. pseudospiralis* with homologous and heterologous CWE

**Fig. 5:**
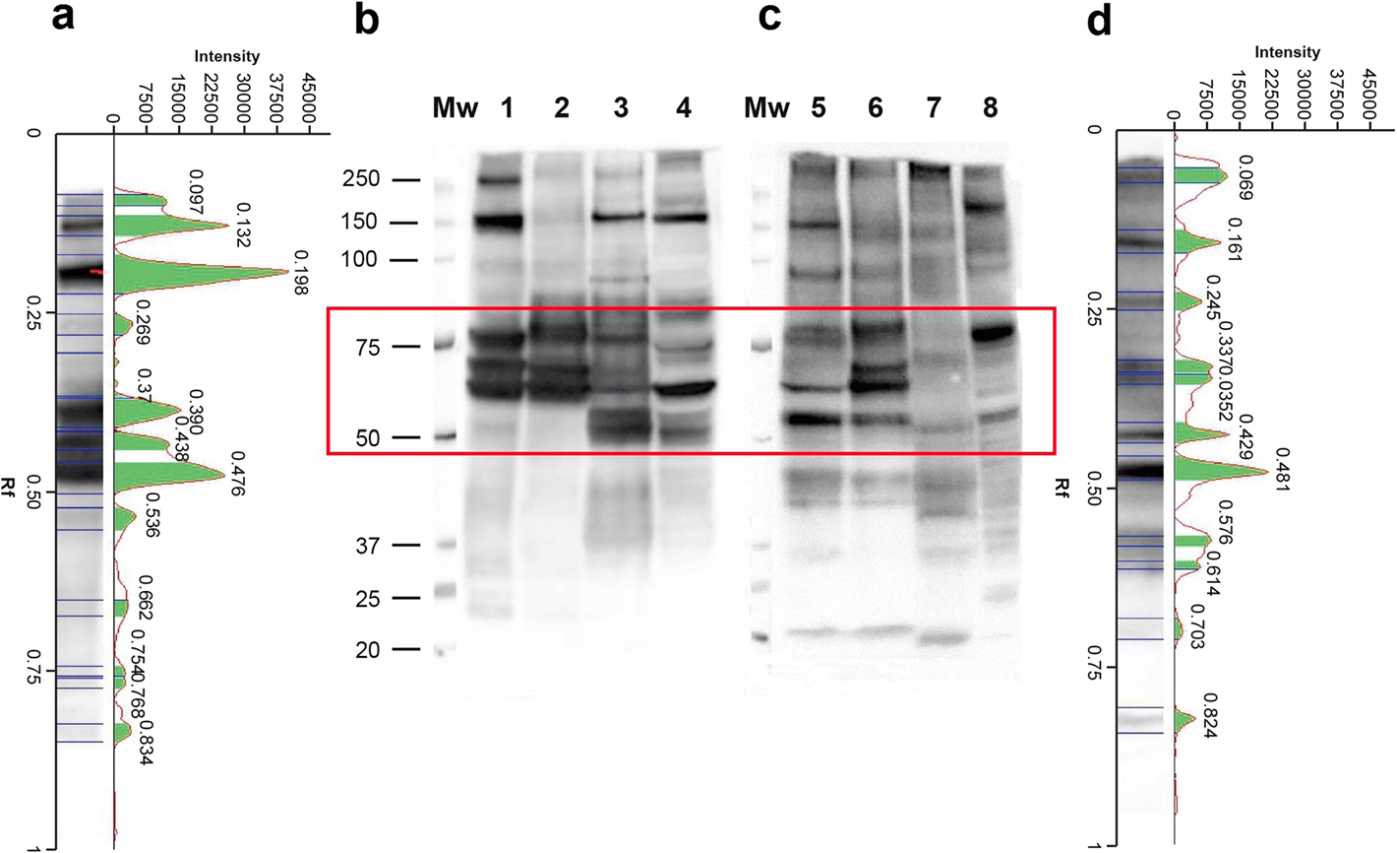
Western blot (Wb) patterns, signal intensities and relative migration values of serum samples from people infected by *Trichinella spiralis* or *T. pseudospiralis*. **a** Signal intensity and relative migration values of *T. spiralis* crude worm extracts (CWE) with a serum sample from a *T. spiralis* infected human serum (**b** Lane 1). **b** Lane Mw, molecular weights in kDa. Wb of *T. spiralis* (Lane 1), *T. britovi* (Lane 2), *T. pseudospiralis* (Lane 3) or *T. papuae* (Lane 4) CWE with anti-*T. spiralis* human serum (Lanes 1–4). **c** Wb of *T. spiralis* (Lane 5), *T. britovi* (Lane 6), *T. pseudospiralis* (Lane 7) or *T. papuae* (Lane 8) CWE with anti-*T. pseudospiralis* human serum (Lanes 5–8). **d** Signal intensity and relative migration values of *T. spiralis* CWE with a serum sample from a *T. pseudospiralis* infected human serum (**c** Lane 5). The red box indicates the highest differences detected in the Wb patterns

Sera from five patients infected with *T. spiralis* and four infected with *T. pseudospiralis* reacted with the CWE of the other species displaying different reactivity patterns (Fig. 5a). For example, the reactivity of *T. spiralis* CWE was qualitatively different when blotted with a *T. spiralis* than with a *T. pseudospiralis* infected serum sample (Fig. 5a, Lane 1; Fig. 5b, Lane 5). The pattern of reactivity of *T. spiralis* CWE with sera from four *T. pseudospiralis-*infected individuals is shown in Fig. 5b, Lanes 1–4. The highest differences were observed from 50 to 75 kDa; the Rf of *T. spiralis* CWE reacting proteins with representative *T. spiralis* and *T. pseudospiralis* human sera were 0.484, 0.448 and 0.397 mm, and 0.486, 0.436, 0.360 and 0.355 mm, respectively (Fig. 5a, red boxes on Lanes 1 and 5).

Human *T. britovi-*infected sera showed the same pattern of reactivity as that of *T. spiralis-*infected patients with *T. spiralis* CWE (data not shown).

## Discussion

The present study demonstrated that western blot using *T. spiralis* CWE can be a useful tool in distinguishing between infections caused by *T. spiralis* or *T. britovi* from those caused by *T. pseudospiralis.* This test is of potential significance for clinical and epidemiological studies, particularly when the source of infection is not traceable. Moreover, since *T. spiralis* is the species generally used for antigen production to detect infections with any species of *Trichinella*, the proposed test will be affordable for most laboratories working in human and animal serology.

Under the experimental conditions used in the present study, a clear pattern of *T. spiralis* CWE reacting proteins able to distinguish between *T. spiralis-*, *T. britovi-* and *T. papuae-*infected sera was not defined. Encapsulated and non-encapsulated species of the genus *Trichinella* diverged from their most recent common ancestor about 21 million years ago (mya), with taxon diversifications commencing 7–10 mya [1]. Biochemical and immunological differences among the species are well documented [20–23]. However, most of these studies focussed on *T. spiralis* and *T. pseudospiralis* showing substantial differences in the inflammatory response and modulation of the muscle cell phenotype [24–31], in larval and adult size [32], naked larva kinesis [14], morphology of the stichocyte granules [33] and protein content [33–38] and therefore highlighted discrepancies in immunological properties [39–42]. Differences in the electrophoretic protein patterns of *T. spiralis* and *T. pseudospiralis* have been reported by several authors. Rodriguez-Perez et al. [43] showed that the electrophoretic reactivity pattern of *T. pseudospiralis* CWE with a monoclonal antibody displayed fewer protein bands than that of *T. spiralis* CWE with the same serum. Wu et al. [33] using homologous murine infected sera revealed 100 and 20–30 antigenic peptide spots in *T. spiralis* CWE and *T. pseudospiralis* CWE, respectively, by two-dimensional Wb, despite the similar level of total protein content in both extracts. These authors concluded that *T. pseudospiralis* has a lower immunogenicity than *T. spiralis* [33]. In the latter study, the immunostaining of *T. pseudospiralis* CWE with homologous murine infected sera were weaker than those of *T. spiralis* CWE with its respective murine infected samples. Our results confirm these differences both in the number of antigenic proteins (bands) and in their reaction intensity (Fig. 2a, c; Figs. 3, 4 and 5), highlighting once more the lower immunogenicity of *T. pseudospiralis* in comparison to that of *T. spiralis* [24, 40, 42, 44].

Differences in pathological changes in the host caused by *T. spiralis* and *T. pseudospiralis* have been attributed to the diversity of their excretory/secretory products [37, 38]. However, it is known that *T. spiralis* and *T. pseudospiralis* share ESA with considerable similarity, with some being identical in terms of cDNA sequence, deducted molecular mass and antigenicity [34, 36, 45, 46]. *Trichinella spiralis* ESA show two main proteins, those migrating at about 43 kDa and those migrating at about 53 kDa [47, 48] and homologous proteins were observed in *T. pseudospiralis*. However, the comparison of the amino acid sequence of the 53 kDa of *T. pseudospiralis* with the corresponding one in *T. spiralis* revealed less than 68% homology. An antibody against the 53 kDa recombinant protein of *T. spiralis* recognized this protein in CWE from adult worms, and in the ESA from *T. spiralis* muscle larvae, but it did not recognize any protein in *T. pseudospiralis* [37, 49]. The results of the present work are based on CWE, which, besides containing ESA, has many more proteins. This protein rich-pool has allowed us to distinguish between *Trichinella* taxa based on the different patterns of reactivity of the native proteins present in the CWEs, with homologous and heterologous sera as described in this study. However, *T. spiralis* ESA can detect infections caused by any of the known *Trichinella* taxa [10], and it is more specific but less sensitive than CWE, having a great utility to differentiate *Trichinella* infections from those caused by other pathogens.

Recently, the immunogenic proteins from somatic muscle larval extracts of *T. spiralis*, *T. pseudospiralis* and *T. papuae* were compared by immunoblotting and mass spectrometry. After immunoblotting with pooled human sera, 17 proteins ranging from 33 to 67 kDa, which were associated with important molecular functions and biological processes of the parasite, were selected for identification. As expected, some proteins were found to be shared among the investigated species whereas other proteins were species-specific [50].

**Fig. 6:**
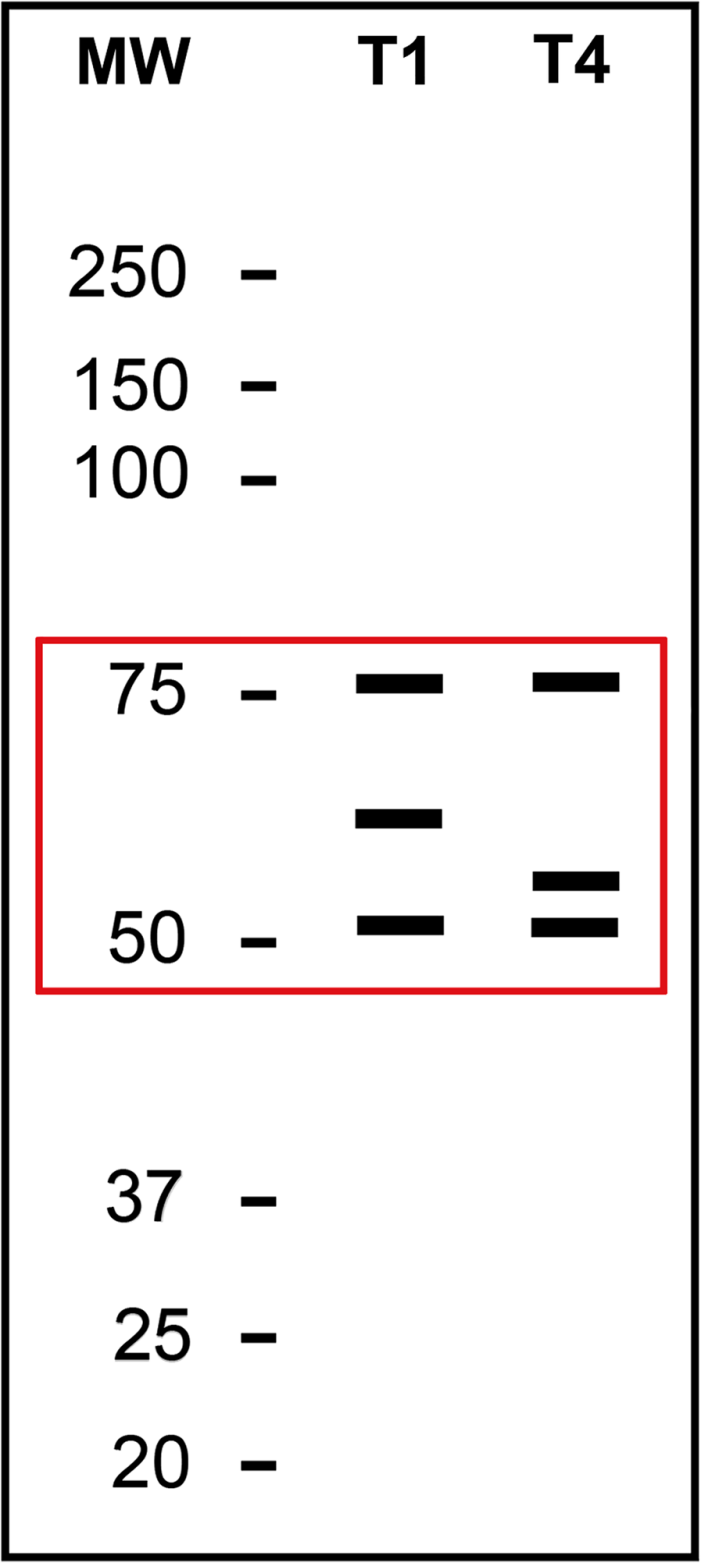
Scheme of the western blot pattern of reactivity of the *Trichinella spiralis* crude worm extract with *T. spiralis* (Lane 1) or *T. pseudospiralis* (Lane 2) sera from infected hosts. Lane Mw: molecular weights in kDa. The red box indicates the highest differences detected in the Wb patterns

Differences observed at the molecular level create a characteristic pattern of reactivity for the native proteins present in the CWEs with sera from different *Trichinella*-infected hosts. This has allowed the inference of the etiological agent (*T. spiralis*/*T. britovi versus T. pseudospiralis*) of infection. The precise determination of the proteins involved in these differences in terms of molecular weight or the relative electrophoretic mobility was not within the scope of this work. However, each serum sample from *T. spiralis*/*T. britovi-* or *T. pseudospiralis-*infected host yielded a “fingerprint” of its own identity when it was blotted with *T. spiralis* CWE (Fig. 6). Under the experimental conditions used in this study, a diagnostic pattern was defined for *T. spiralis*/*T. britovi-* and *T. pseudospiralis-*infected hosts.

The present results may be useful in the epidemiological context of the European Union, but some of the experiments using *T. papuae* clearly show the potential of this diagnostic method in differentiating between *Trichinella* species infecting animals and humans from Southeast Asia, where *T. spiralis* and *T. papuae* occur sympatrically. Wb of *T. spiralis* CWE with serum samples from mice infected with all the 12 *Trichinella* taxa recognized so far, show similar protein patterns excluding the *T. pseudospiralis-*infected mouse serum, which can be easily distinguished by other patterns (Additional file 1: Figure S1). These Wb patterns also show several different bands; however, additional investigation carried out with serum samples from other host species are required to study these differences and to use them as diagnostic tools.

## Conclusions

The present study suggests that Wb using *T. spiralis* CWE may be a useful tool to distinguish *Trichinella* infections caused by *T. pseudospiralis* from those caused by *T. spiralis* or *T. britovi*. This method may support epidemiological investigations, particularly when the source of infection is not traceable.

## Additional file

Additional file 1: **Figure S1.** Western blot (Wb) patterns of reactivity of *Trichinella spiralis* crude worm extract with sera from mice infected with *T. spiralis* (T1), *T. nativa* (T2), *T. britovi* (T3), *T. pseudospiralis* (T4), *T. murrelli* (T5), *Trichinella* T6, *T. nelson*i (T7), *Trichinella* T8, *Trichinella* T9, *T. papuae* (T10) *T. zimbabwensis* (T11) and *T. patagoniensis* (T12). Lane molecular weights (Mw) are in kDa. (TIF 959 kb)

## Abbreviations

CWE: crude worm extract
dpi: days post-infection
ELISA: enzyme linked immunosorbent assay
ESA: excretory/secretory antigens
IgG: Immunoglobulin G
ML: muscle larvae
Mw: molecular weight
mya: million years ago
OD: optical density
PBS: phosphate-buffered saline
Rf: relative mobility
RT: room temperature
SDS-PAGE: sodium dodecyl sulphate polyacrylamide gel electrophoresis
TBST: Tris-borate saline Tween
Wb: western blot
