# Supplementary figures and images for: Differentiation of Trichinella species (Trichinella spiralis/Trichinella britovi versus Trichinella pseudospiralis) using western blot
Source: Parasit Vectors. 2018 Dec 12;11:631. doi: 10.1186/s13071-018-3244-3 (PMC6291991; doi:10.1186/s13071-018-3244-3)

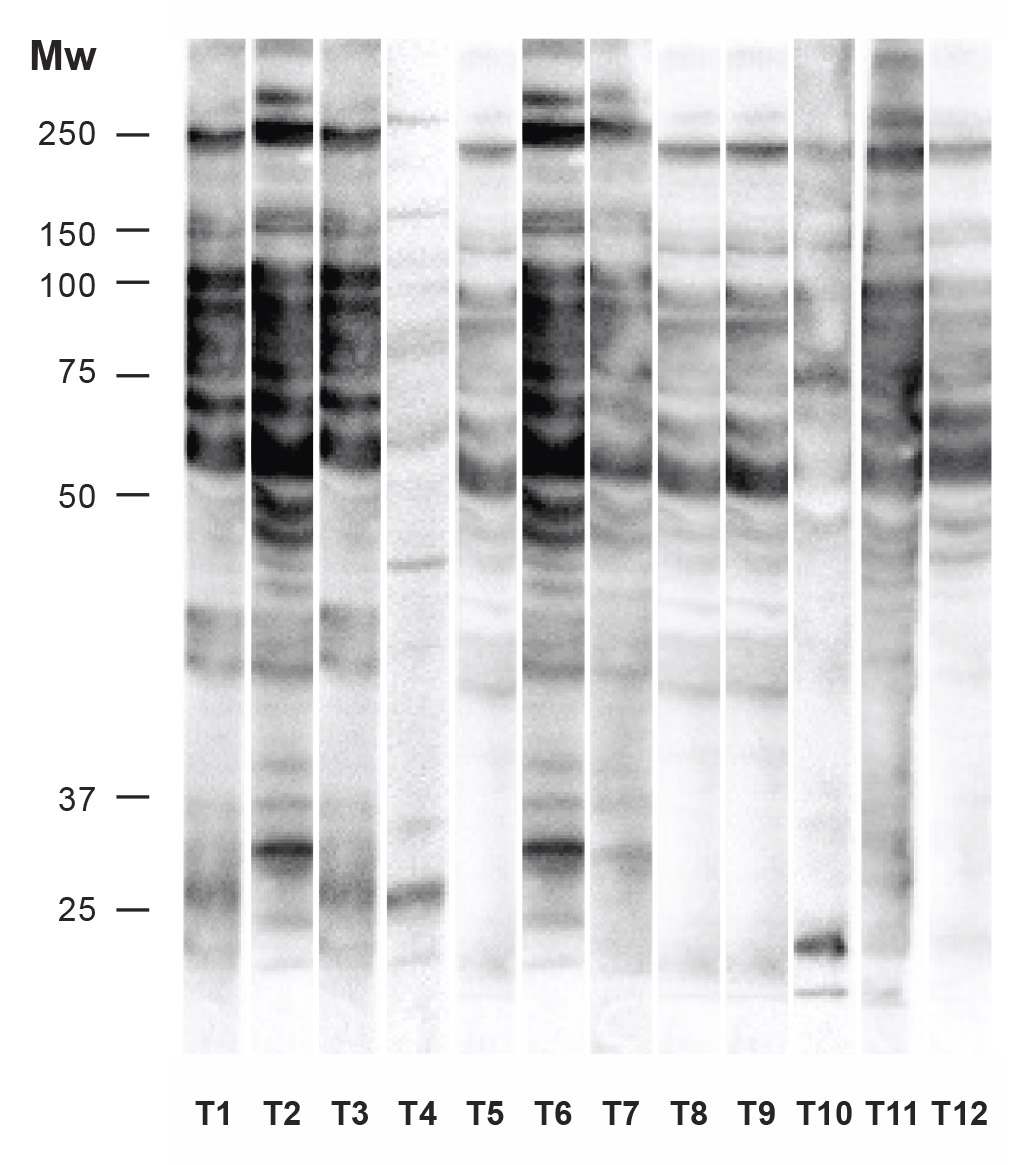

Supplement: Supplementary file 1 — Figure S1. Western blot (Wb) patterns of reactivity of Trichinella spiralis crude worm extract with sera from mice infected with T. spiralis (T1), T. nativa (T2), T. britovi (T3), T. pseudospiralis (T4), T. murrelli (T5), Trichinella T6, T. nelsoni (T7), Trichinella T8, Trichinella T9, T. papuae (T10) T. zimbabwensis (T11) and T. patagoniensis (T12). Lane molecular weights (Mw) are in kDa. (TIF 959 kb) [file 13071_2018_3244_MOESM1_ESM.tif]
